# LINC01559 drives osimertinib resistance in NSCLC through a ceRNA network regulating miR-320a/IGF2BP3 axis

**DOI:** 10.3389/fphar.2025.1592846

**Published:** 2025-04-17

**Authors:** Leidi Xu, Yibo Zhang, Liangliang Xing, Ying Zhou, Ning Chang, Hangtian Xi, Xiangrui Xu, Jian Zhang

**Affiliations:** ^1^ Department of Pulmonary and Critical Care Medicine, Xijing Hospital, Air-Force Medical University, Xi’an, China; ^2^ Department of Pulmonary Medicine, Chest Hospital in Xi’an People’s Hospital, Xi’an, Shaanxi, China

**Keywords:** LINC01559, osimertinib resistance, lung adenocarcinoma, ceRNA network, miR-320a/IGF2BP3 axis

## Abstract

**Background:**

Osimertinib resistance remains a major challenge in the treatment of lung adenocarcinoma. Long non-coding RNAs (lncRNAs) have emerged as key regulators of drug resistance, but their roles in osimertinib resistance are poorly understood. This study aimed to identify lncRNAs driving osimertinib resistance and elucidate their molecular mechanisms.

**Methods:**

Multi-cohort analysis (GSE222820, GSE232890, GSE255958) identified osimertinib resistance-associated lncRNAs. Functional validation employed *in vitro* assays (proliferation, migration, invasion, drug sensitivity) and xenograft models. Mechanistic studies involved luciferase reporter assays, RNA immunoprecipitation (RIP), and Western blotting. Clinical correlations were analyzed using TCGA-LUAD data.

**Results:**

Our findings demonstrated that LINC01559 was markedly upregulated in LUAD tissues and osimertinib-resistant cell lines, correlating with poor patient survival. Functional analyses revealed that LINC01559 critically regulates processes linked to drug resistance, enhancing tumor cell proliferation, migration, and invasive capabilities. Knockdown of LINC01559 sensitized resistant cells to osimertinib, significantly reducing colony-forming potential and suppressing migratory/invasive behaviors. In contrast, overexpression of LINC01559 exacerbated therapeutic resistance. Mechanistically, LINC01559 functions as a competing endogenous RNA (ceRNA) by sponging miR-320a, promote osimertinib -resistance and upregulate the expression of the miR-320a target IGF2BP3. Rescue experiments and xenograft models confirmed that Linc01559 drives resistance via the miR-320a/IGF2BP3 axis.

**Conclusion:**

This study identifies LINC01559 as a novel ceRNA that drives osimertinib resistance in lung adenocarcinoma by sponging miR-320a to enhance IGF2BP3 expression. Targeting the LINC01559/miR-320a/IGF2BP3 axis may provide a therapeutic strategy to overcome osimertinib resistance.

## Introduction

Lung cancer remains a leading cause of cancer-related mortality worldwide, with around 2.2 million new cases and 1.8 million deaths occurring annually (Sung et al., 2021). The majority of lung cancers (85%) are non-small cell lung cancer (NSCLC), which is frequently diagnosed at advanced stages; yet nearly 30% of individuals are diagnosed with early-stage (I–III) disease that is resectable (Goldstraw et al., 2016). Epidermal growth factor receptor (EGFR) mutations, pivotal oncogenic drivers in NSCLC, are detected in 30%–50% of Asian and 10%–20% of Western populations (Midha et al., 2015; Shi et al., 2014). Recent EARLY-EGFR study revealed a 51% EGFR mutation rate in resected stage I–III NSCLC, emphasizing the clinical relevance of molecular profiling even in localized tumors. These mutations make cells sensitive to EGFR tyrosine kinase inhibitors (TKIs), which have revolutionized treatment paradigms in advanced NSCLC (Tsuboi et al., 2023).

While osimertinib, as the third-generation TKI, demonstrates superior efficacy against both sensitizing and resistant T790M mutations, acquired resistance remains inevitable, with emerging mechanisms broadly categorized as on-target EGFR alterations and off-target bypass signaling activation (Fu et al., 2022). Current investigations point out the vital importance of epigenetic irregularities in osimertinib resistance, including DNA hypermethylation of tumor suppressor genes and histone modification-driven activation of pro-survival pathways (Mabe et al., 2024). As critical components of epigenetic regulation, lncRNAs remain understudied in osimertinib resistance, urging systematic investigations to delineate their roles in EGFR-mutant NSCLC.

Long non-coding RNAs (lncRNAs) are a class of non-protein-coding transcripts exceeding 200 nucleotides in length, which regulate gene expression at epigenetic, transcriptional, and post-transcriptional levels (Lee, 2012). Unlike mRNAs, lncRNAs exhibit low sequence conservation but play pivotal roles in diverse biological processes, including chromatin remodeling, enhancer-promoter looping, and RNA splicing (Nojima and Proudfoot, 2022). Notably, their dysregulation is closely associated with pathological states, particularly cancer. For instance, the imprinted lncRNA PVT1 stabilizes MYC oncogene expression by competing for enhancer access, while XIST orchestrates X-chromosome inactivation through recruitment of repressive chromatin modifiers (Shigeyasu et al., 2020; Dror et al., 2024). A recent study suggests LOC85009, can modulate drug sensitivity in lung adenocarcinoma by regulating autophagy (Yu et al., 2023). In cancer progression, lncRNAs act as dynamic regulators of drug resistance. They modulate chemosensitivity by altering DNA repair efficiency, activating survival pathways, or interacting with drug transporters (Adriaens et al., 2016; Chen et al., 2025; Wang et al., 2020; Tan et al., 2024). A prominent mechanism involves the competitive endogenous RNA (ceRNA) network, where lncRNAs sequester microRNAs (miRNAs) through complementary binding sites, thereby derepressing miRNA-targeted mRNAs (Karreth and Pandolfi, 2013). For example, the progression of gastric cancer is inhibited by LINC01133, which functions as a ceRNA by sequestering miR-106a-3p, thereby restoring APC expression and suppressing oncogenic Wnt/β-catenin signaling (Yang et al., 2018).

As one of the substrates of lncRNA, MicroRNAs, or miRNAs, are short RNA sequences that do not code for proteins and suppress gene expression by binding to specific mRNAs after transcription. (Fabian and Sonenberg, 2012). Last year’s The Nobel Prize in Physiology or Medicine was given to Victor Ambros and Gary Ruvkun for their identification of miRNA and its involvement in regulating transcription (Lee et al., 1993; Calin et al., 2024). miRNAs have been confirmed to regulate multiple biological processes, including embryonic development, cell differentiation, immune modulation, and tumor progression. For example, miR-302 maintains the pluripotency of stem cells by suppressing differentiation-related genes (Li et al., 2016), while miR-21 promotes cancer metastasis by downregulating Genes that suppress tumors, like PTEN and PDCD (Melnik, 2015). Beyond their roles in tumor progression, miRNAs are increasingly recognized as pivotal regulators of chemoresistance through diverse mechanisms, such as miR-34a enhances cisplatin sensitivity by focusing on ABCB1-dependent pathways for drug efflux in ovarian cancer (Li et al., 2014), and miR-155 drives resistance to 5-fluorouracil in colorectal cancer by suppressing DNA repair mechanisms (Hess et al., 2017). Despite these advances, miRNA-mediated mechanisms in osimertinib resistance remain poorly characterized.

IGF2BP3, part of the conserved family of RNA-binding proteins, is known as insulin-like growth factor 2 mRNA-binding protein 3, plays critical roles in post-transcriptional gene regulation through its six KH domains that recognize specific RNA motifs (Yang et al., 2023). Emerging evidence highlights IGF2BP3 as a key epigenetic regulator through its dual capacity to recognize both N6-methyladenosine (m6A) and internal N7-methylguanosine (m7G) modifications on target mRNAs (Shan et al., 2023; Liu et al., 2024). As an m6A reader, IGF2BP3 stabilizes methylated transcripts like MCM5 to activate oncogenic signaling pathways, while its interaction with m7G-modified mRNAs promotes their degradation through exosome-mediated decay. Recent studies reveal that post-translational modifications of IGF2BP3 itself, particularly lysine lactylation driven by glycolytic reprogramming, enhance its RNA-binding activity and create feedforward loops that sustain antioxidant defenses in hepatocellular carcinoma (Lu et al., 2024). Another study demonstrates IGF2BP3 stabilizes COX6B2 mRNA via m6A modification at its 3′UTR, inducing resistance to EGFR-TKI in NSCLC by enhancing OXPHOS and nicotinamide metabolism (Lin et al., 2023). While these findings underscore IGF2BP3’s multifaceted roles in RNA metabolism and therapy resistance, the upstream regulatory networks orchestrating its activity—particularly ncRNA-mediated control—remain elusive in the context of osimertinib resistance. Here we report that LINC01559, a previously uncharacterized lncRNA, drives osimertinib resistance by functioning as a miR-320a sponge to stabilize IGF2BP3 and its downstream oncogenic effectors. Through integrated multi-omics analyses, functional validation, and *in vivo* modeling, we demonstrate that the LINC01559/miR-320a/IGF2BP3 axis promotes osimertinib resistance. Our study uncovers a novel ncRNA-dependent epigenetic circuit governing IGF2BP3-driven osimertinib resistance, providing actionable targets to reverse TKI tolerance in LUAD.

## Methods and materials

### Cell culture and drug-resistant cell line establishment

Cell lines A549, H1299, H1975, PC9, HCC827, HEK-293t, and BEAS-2B were sourced from the Chinese Academy of Sciences (CAS, Shanghai, China), and all were cultured in RPMI-1640 (H1975, PC9, HCC827) or DMEM (A549, H1299, BEAS-2B, HEK293T) supplemented with 10% fetal bovine serum (FBS) and maintained at 37°C in 5% CO_2_.

Osimertinib-resistant PC9 (PC9/OR) and H1975 (H1975/OR) sublines were established by continuous exposure to escalating osimertinib (S7297, Selleck) concentrations from 10 nM to 1 μM over 6 months. Drug resistance stability was confirmed by maintaining cells in drug-free medium for ≥2 weeks without reversal of phenotypic properties.

### Plasmid, oligonucleotides, cell transfection and virus infection

The LINC01559 overexpression plasmid (GV658 vector) and lentiviral particles encoding two independent shRNAs targeting LINC01559 (sh1: 5′-CCT​AAA​GAG​TTG​GCA​CTC​TAT-3’; sh2: 5′- TCA​AAC​CAG​GTG​CAA​TTA​TTT-3′) were purchased from Hanheng (Shanghai, China). For transient overexpression, the plasmid was introduced into cells with Lipofectamine 3000 (Thermo Fisher Scientific) as per the manufacturer’s guidelines, and the efficiency of transfection was verified through qRT-PCR. For stable knockdown, cells were infected with shRNA lentiviruses for 48 h, followed by puromycin selection. miRNA mimics (miR-320a) and inhibitors (anti–miR-320a) were purchased from Hanheng (Shanghai, China) and transiently transfected for 48 h using Lipofectamine 3000 (Invitrogen).

### Western blot

Cells were lysed in RIPA buffer (Beyotime, Cat# P0013B) supplemented with protease inhibitors (Roche, Cat# 04693159001). Protein concentrations were quantified using a BCA Protein Assay Kit (Thermo Fisher Scientific, Cat# 23225). Equal amounts of protein (20–30 μg) were separated by 10% SDS-PAGE and transferred onto PVDF membranes (Millipore, Cat# IPVH00010). Blotting membranes were initially blocked with 5% skim milk prepared in Tris-buffered saline containing 0.1% Tween-20 (TBST) for 2 h at ambient temperature. Membranes were subsequently incubated overnight with primary antibodies at 4°C. Following three washes with TBST, membranes were incubated with horseradish peroxidase (HRP)-linked secondary antibodies (Proteintech; 1:5000 dilution) for 1 h at room temperature. Protein bands were visualized using an enhanced chemiluminescence (ECL) detection system (Mishu Shengwu, Cat# MI00607B) and quantified with ImageJ software (version 1.8.0). The following primary antibodies were used: CD44 (1:5000, Proteintech, Cat# 15675-1-AP); IGF2BP3 (1:1000, Proteintech, Cat# 14642-1-AP); c-MYC (1:2000, Proteintech, Cat# 10828-1-AP); GAPDH (1:5000, Proteintech, Cat# 60004-1-Ig).

### Quantitative RT–PCR

Total RNA was extracted using TRIzol reagent (Invitrogen, Cat# 15596026), with RNA purity (A260/A280: 1.8–2.0) confirmed using a NanoDrop 2000 (Thermo Fisher Scientific). For mRNA analysis, 1 μg of total RNA was reverse-transcribed into cDNA using the PrimeScript RT Reagent Kit (Takara, Cat# RR047A). miRNA-specific cDNA was synthesized with stem-loop primers (RiboBio) and the Mir-X miRNA First-Strand Synthesis Kit (Takara, Cat# 638315). Quantitative PCR was performed on a QuantStudio 5 system (Applied Biosystems) using SYBR Green Master Mix (Roche, Cat# 4913914001) under standard cycling conditions. Relative gene expression was calculated using the 2^−ΔΔCT^ method. Statistical significance (p < 0.05) was determined using Student’s t-test or ANOVA in GraphPad Prism 9.0, with three independent replicates performed. Primer sequences are provided in Supplementary Table S1.

### Cell viability assay

PC-9, H1975, and transfected cells were seeded into 96-well plates (2 × 10^3^ cells/well). Cells were treated with osimertinib or vehicle for 72 h. Cell viability was monitored using the CCK-8 assay (Dojindo Molecular Technologies, Kumamoto, Japan) following the manufacturer’s protocol. Absorbance was measured at 450 nm at 24, 48, 72, and 96 h. The half-maximal inhibitory concentration (IC_50_) was calculated via nonlinear regression analysis using GraphPad Prism (V9.0).

### Colony formation assay

For colony formation, transfected cells were seeded into 6-well plates at a density of 500 cells/well and cultured for 14 days. Colonies were fixed with 4% paraformaldehyde (Biosharp, Cat# BL539A), stained with crystal violet (Beyotime, Cat# C0121), and washed gently with PBS. Colonies were counted using ImageJ (V1.8.0)and analyzed by GraphPad Prism (V9.0).

### Wound healing assay

For wound healing assay, transfected cells were seeded into 6-well plates (5 × 10^5^ cells/well). A sterile 200 μL pipette tip was used to create a linear scratch in the cell monolayer. Detached cells were removed by washing with PBS, and fresh serum-free medium was added. Wound closure was monitored at 0 h and 24 h using an inverted phase-contrast microscope (Nikon Eclipse Ti2). Migration distance was quantified by measuring the residual wound area at each time point using ImageJ (V1.8.0).

### Transwell assay

Cell migration and invasion were assessed using uncoated or Matrigel-coated Transwell chambers (8-μm pores; Corning, Cat# 3422), respectively. For invasion assays, 1 × 10^5^ cells in serum-free medium were seeded into Matrigel-coated upper chambers, while migration assays used uncoated inserts with 5 × 10^4^ cells. After 24 h incubation, non-migrated/invaded cells were removed, and cells on the lower membrane were fixed (4% paraformaldehyde), stained (0.1% crystal violet), and quantified in five random fields (200×). The number of cells traversing the pores was quantified using ImageJ (V1.8.0).

### Nuclear-cytoplasmic fractionation

Subcellular RNA fractionation was performed to isolate nuclear and cytoplasmic RNA components using the NE-PER™ Kit (Thermo Fisher Scientific, Cat# 78833). Briefly, transfected cells (5 × 10^6^) were harvested, washed with phosphate-buffered saline (PBS), and homogenized in pre-chilled cytoplasmic extraction reagents (CER I/II) according to the manufacturer’s guidelines to partition cytoplasmic contents. The remaining nuclear pellet was resuspended in nuclear extraction reagent (NER) and vortexed vigorously. RNA from both fractions was purified using TRIzol and stored at −80°C.

### RNA immunoprecipitation

RIP assays were performed using the Magna RIP™ Kit (Millipore 17–700). Briefly, cells were lysed in RIP buffer containing protease/RNase inhibitors and incubated overnight at 4°C with 5 μg Anti-AGO2 antibody (Abcam ab57113) or normal mouse IgG (Millipore, 12–371) as negative control. RNA-protein complexes were captured using Protein A/G magnetic beads, followed by RNA extraction with TRIzol. Enrichment of LINC01559 and miR-320a in immunoprecipitates was quantified via qPCR.

### Luciferase reporter assays

For cells (1 × 10^5^ cells/well) were seeded in 24-well plates. Wild-type or mutant LINC01559 fragments and IGF2BP3 3′-UTR sequences were synthesized by Hanheng Biotechnology and cloned into the pmirGLO dual-luciferase vector. Cells were co-transfected with 200 ng reporter plasmid and 50 nM miR-320a mimics or negative control using Lipofectamine 3000. After 48 h, following cell lysis with Promega’s Passive Lysis Buffer Firefly and Renilla luciferase signals were sequentially quantified using a Dual-Luciferase Assay Kit (Promega) on a microplate luminometer. Relative luciferase activity (Firefly/Renilla ratio) was normalized to controls.

### Animal experiments

Female NU/NU nude mice (6 weeks old, Charles River) were randomized into four groups (n = 6/group) and housed under specific pathogen-free conditions. H1975 cells (5 × 10^6^) stably expressing shControl or sh1-LINC01559 were subcutaneously injected into the right axilla. Osimertinib (5 mg/kg) or vehicle was administered daily via oral gavage starting on Day 14 post-inoculation. Tumor dimensions were measured every 4 days using calipers, with volume calculated as $\frac{\text{length}\times {\text{width}}^{2}}{2}$ . Mice were sacrificed at Day 30, and tumors were excised and weighed. All procedures were approved by the Animal Ethics Committee of Air Force Medical University (No 20240605) and complied with ARRIVE guidelines.

### Bioinformatics analysis and target gene prediction

Differentially expressed lncRNAs (DELs) in osimertinib-resistant cells and osimertinib-sensitive cells were identified from three public transcriptomic datasets (GSE222820, GSE232890, GSE255958) using DESeq2 and limma R package (|log2 fold change| >1, adjusted *p* < 0.05). To delineate biological functions and pathways associated with differentially expressed lncRNAs (DELs), we conducted Gene Ontology (GO), KEGG, and Gene Set Enrichment Analysis (GSEA) via the R package clusterProfiler. Statistically significant terms (nominal *p* < 0.05, false discovery rate <0.1) were identified to characterize DEL-related molecular mechanisms. Survival analysis of Linc01559 in lung adenocarcinoma (LUAD) patients was conducted using TCGA-LUAD cohort data, with Kaplan-Meier curves and log-rank tests to assess prognostic significance. Potential miRNAs (miR-320a) interacting with Linc01559 were predicted via TargetScan (V8.0). Putative miR-320a targets were predicted using seven miRNA databases. Consensus targets were defined as mRNAs identified in at least six databases, visualized via the *upset* R package. These candidates were intersected with differentially expressed mRNAs (|log2FC| >1, adjusted *p* < 0.05) from three transcriptomic datasets above. IGF2BP3 was identified as the sole overlapping gene across all filters.

### Statistical analysis

Data are presented as mean ± standard deviation (SD) from at least three independent experiments. Statistical analyses were performed using GraphPad Prism 9.0 and R (v4.3.1). Two-group comparisons were analyzed by unpaired Student’s *t*-test. Multiple group comparisons were assessed by one-way ANOVA. Survival curves were generated via Kaplan-Meier analysis, and significance was determined by the log-rank test. Dose-response curves (IC50) were fitted using nonlinear regression. Gene set enrichment analysis (GSEA) significance thresholds were set at nominal *p* < 0.05 and FDR <0.1. Statistical significance was denoted as follows: *p < 0.05, **p < 0.01, and ***p < 0.001.

## Results

### LINC01559 identified as a core osimertinib resistance-associated lncRNA

**FIGURE 1:**
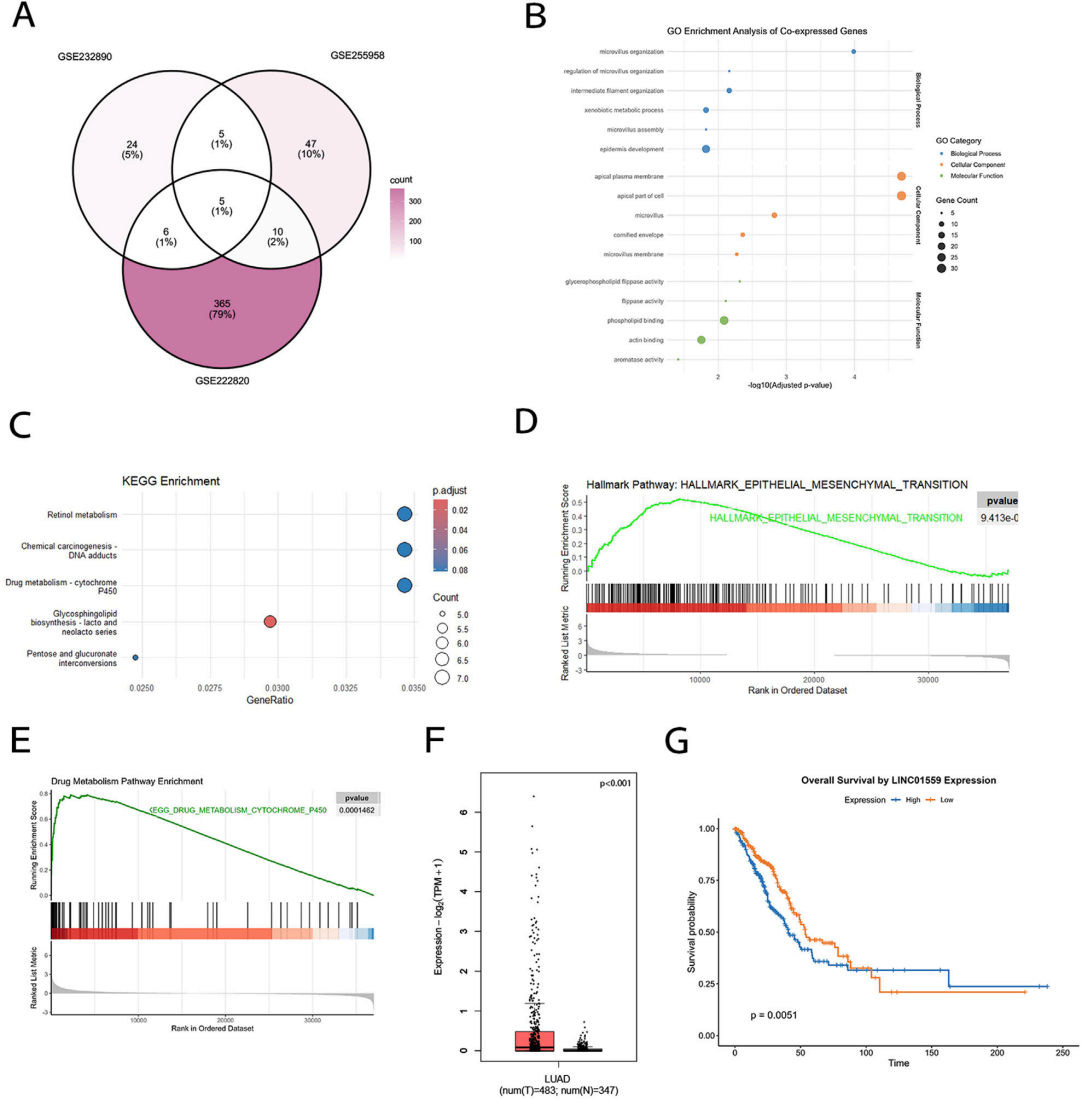
LINC01559 is a core osimertinib resistance-associated lncRNA in LUAD. **(A)** Venn diagram showing five overlapping lncRNAs across three osimertinib-resistant LUAD datasets (GSE222820, GSE232890, GSE255958). LINC01559 exhibited the most significant dysregulation. **(B)** GO analysis of LINC01559 co-expressed genes, highlighting enrichment in microtubule organization and extracellular matrix remodeling. **(C)** KEGG pathway analysis linking LINC01559 to retinol metabolism and xenobiotic detoxification. **(D, E)** GSEA showing significant enrichment of EMT (FDR q < 0.001) and xenobiotic metabolism (FDR q < 0.01) pathways in LINC01559-high tumors. **(F)** LINC01559 expression in TCGA-LUAD tumors versus adjacent normal tissues. **(G)** Kaplan-Meier survival analysis demonstrating shorter overall survival in LUAD patients with high LINC01559 expression. Data are presented as mean ± SD.

To investigate lncRNAs associated with osimertinib drug resistance, we analyzed three independent osimertinib-resistant and -sensitive LUIAD datasets (GSE222820, GSE232890, GSE255958). Venn diagram analysis identified 5 overlapping lncRNAs across all datasets among which LINC01559 exhibited the most significant dysregulation in resistant cell lines (Figure 1A). To elucidate LINC01559’s functional role, Gene Ontology (GO) analysis of its co-expressed protein-coding genes highlighted enrichment in microtubule organization and extracellular matrix remodeling processes implicated in drug efflux and tumor microenvironment adaptation (Figure 1B). KEGG pathway analysis further linked LINC01559 to retinol metabolism and xenobiotic detoxification (Figure 1C), key pathways governing tyrosine kinase inhibitor (TKI) resistance. Gene Set Enrichment Analysis (GSEA) further corroborated LINC01559’s mechanistic role, revealing significant enrichment of epithelial-mesenchymal transition (EMT; FDR *q* < 0.001) and xenobiotic metabolism pathways (FDR *q* < 0.01) in LINC01559-high tumors (Figures 1D, E), aligning with its dual role in metastatic progression and therapeutic adaptation. To evaluate clinical relevance, we analyzed LINC01559 expression in the TCGA-LUAD cohort. Tumor tissues exhibited an elevation in LINC01559 levels compared to adjacent normal tissues (Figure 1F). Critically, Kaplan-Meier survival analysis demonstrated that LUAD patients with high LINC01559 expression had significantly shorter overall survival (Figure 1G), establishing its prognostic value in osimertinib-resistant disease.

### LINC01559 promotes cell proliferation, migration, invasion and osimertinib resistance

**FIGURE 2:**
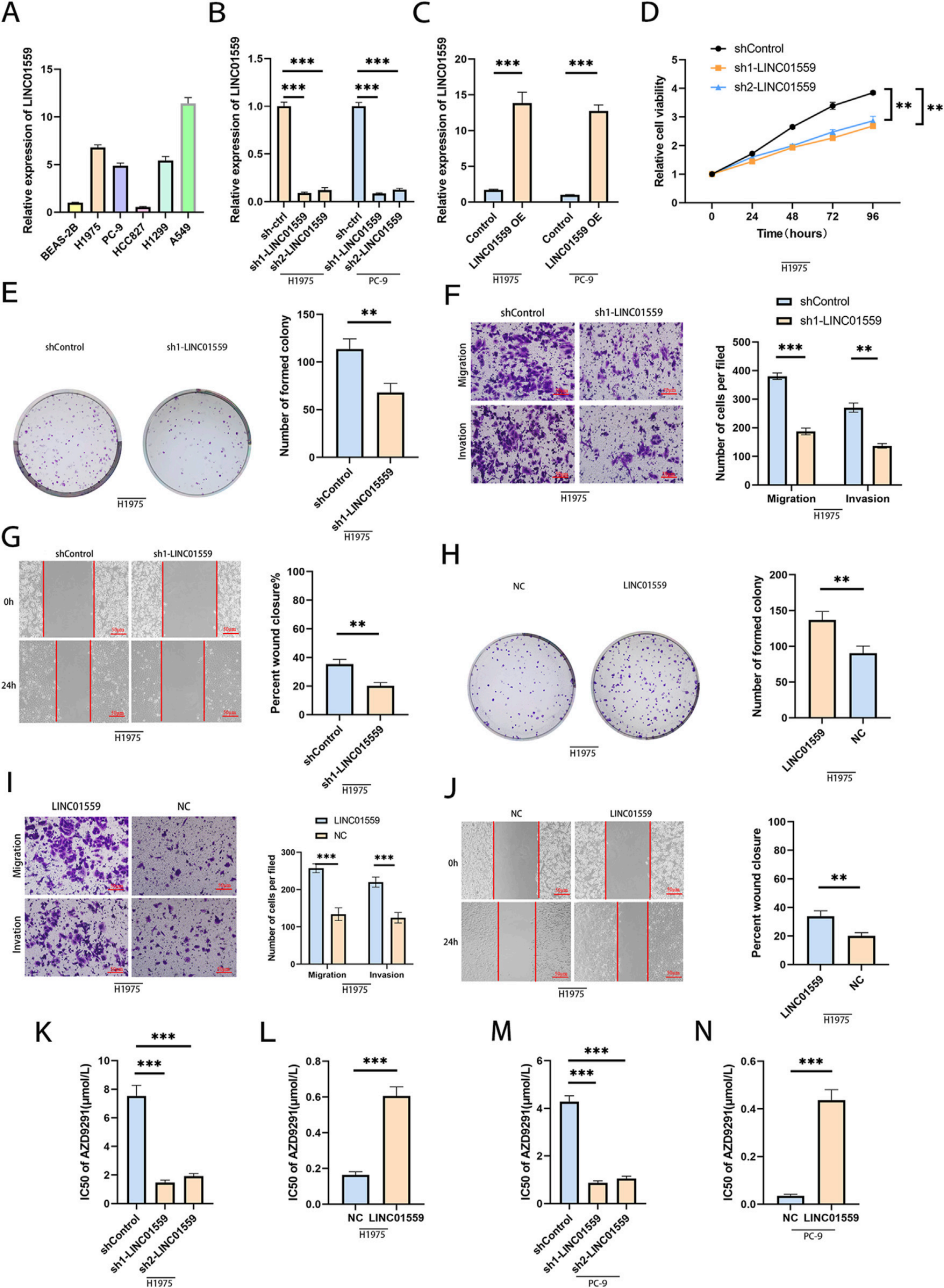
LINC01559 promotes malignant phenotypes and osimertinib resistance. **(A)** LINC01559 expression in lung cancer cell lines (H1975, PC-9) versus normal bronchial epithelial cells (BEAS-2B). **(B, C)** Validation of LINC01559 knockdown (KD) and overexpression (OE) efficiency in H1975 and PC-9 cells. **(D–G)** Functional assays showing reduced proliferation (CCK-8), colony formation (crystal violet), migration, and invasion (Transwell) in LINC01559-KD cells. **(H–J)** Enhanced proliferation, migration, and invasion in LINC01559-OE cells. **(K–N)** Osimertinib sensitivity assays: KD sensitized resistant cells (IC50 reduction), while OE increased resistance (IC50 elevation). Data are mean ± SD; *p < 0.05, **p < 0.01, ***p < 0.001 (two-way ANOVA).

To investigate the biological functions of LINC01559, we first analyzed its expression across lung cancer cell lines and normal bronchial epithelial cells (Figure2A). The results showed LINC01559 was barely detectable in BEAS-2B and HCC827, while its expression varied significantly in other lung cancer cells. Notably, H1975 and PC-9 commonly used osimertinib-resistant cell lines exhibited relatively high LINC01559 levels (*p < 0.001* vs BEAS-2B), thus selected for the subsequent stable knockdown (KD) and overexpression (OE) experiments.

To validate LINC01559’s role in lung cancer, we established stable knockdown and overexpression in H1975 and PC-9 cells. Transfection efficiency was confirmed by substantial reduction in LINC01559 expression in knockdown groups (Figure 2B) and marked upregulation in overexpression groups (Figure 2C). Functional assays revealed that LINC01559 knockdown significantly suppressed proliferation (Figure 2D), reduced colony formation (Figure 2E), and inhibited migration and invasion (Figure 2F). Conversely, overexpression robustly enhanced these malignant phenotypes (Figures 2H, I). Wound healing assays further supported these findings, showing delayed scratch closure in knockdown groups (Figures 2G, J). Critically, silencing LINC01559 markedly sensitized resistant cells to osimertinib (Figures 2K, L), while overexpression increased drug resistance (Figures 2M, N). Together, these results demonstrate that LINC01559 drives tumor growth, metastasis, and osimertinib resistance in lung cancer.

### LINC01559 acts as a sponge of miR-320a

**FIGURE 3:**
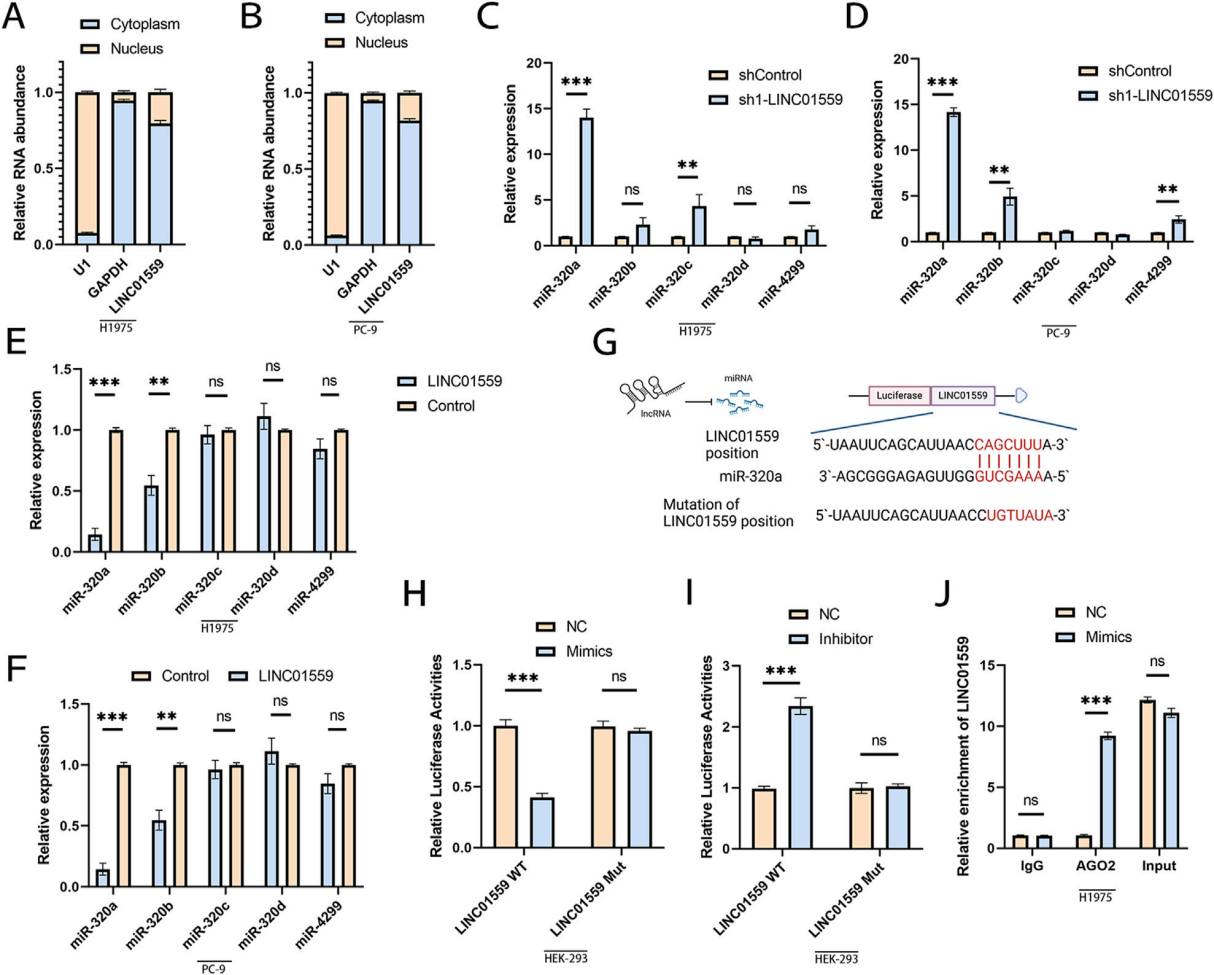
LINC01559 acts as a ceRNA by sponging miR-320a. **(A, B)** Nuclear-cytoplasmic fractionation confirming LINC01559’s predominant cytoplasmic localization. **(C–F)** qPCR showing inverse correlation between LINC01559 and miR-320a expression in KD/OE cells. **(G)** Predicted miR-320a binding sites in LINC01559 and mutant (Mut) constructs. **(H, I)** Dual-luciferase reporter assays: miR-320a mimics suppressed WT-LINC01559 activity, while inhibitors rescued it. **(J)** RIP assay demonstrating LINC01559 enrichment in AGO2 complexes. Data are mean ± SD; *p < 0.05, **p < 0.01, ***p < 0.001 (Student’s t-test).

To investigate the molecular mechanism of LINC01559, we first determined its subcellular localization. Nuclear-cytoplasmic fractionation assays revealed that LINC01559 was mainly localized in the cytoplasm (Figures 3A, B), suggesting its potential role as a competing endogenous RNA (ceRNA) to regulate miRNA activity.

Based on this observation, we used TargetScan to predict miRNA binding sites and identified predicted five candidate miRNAs with potential binding sites on LINC01559. To validate this interaction, we analyzed miRNA expression in LINC01559-modulated cells. Knockdown of LINC01559 significantly upregulated miR-320a levels (Figure 3C), whereas overexpression markedly suppressed miR-320a expression (Figure 3E), Moderate but significant differences were also observed for miR-320b. Similar trends were observed in PC-9 cells (Figures 3D, F).

To further validate the interaction between LINC01559 and miR-320a, we performed dual-luciferase reporter assays using wild-type (WT) LINC01559 and binding site-mutated (Mut) constructs (Figure 3G). Co-transfection of miR-320a mimics with WT LINC01559 significantly suppressed luciferase activity (Figure 3H), whereas mutation of the miR-320a binding site (Mut) abolished this repression. Conversely, miR-320a inhibition rescued luciferase activity in WT LINC01559-transfected cells (Figure 3I), confirming sequence-specific binding. RNA immunoprecipitation (RIP) assays demonstrated significant enrichment of LINC01559 in AGO2 complexes compared to IgG controls (Figure 3J), consistent with its recruitment into miRNA-mediated regulatory pathways. Together, these findings demonstrate that LINC01559 functions as a ceRNA to sequester miR-320a, thereby promoting osimertinib resistance.

### MiR-320a directly targets IGF2BP3 to regulate osimertinib resistance

**FIGURE 4:**
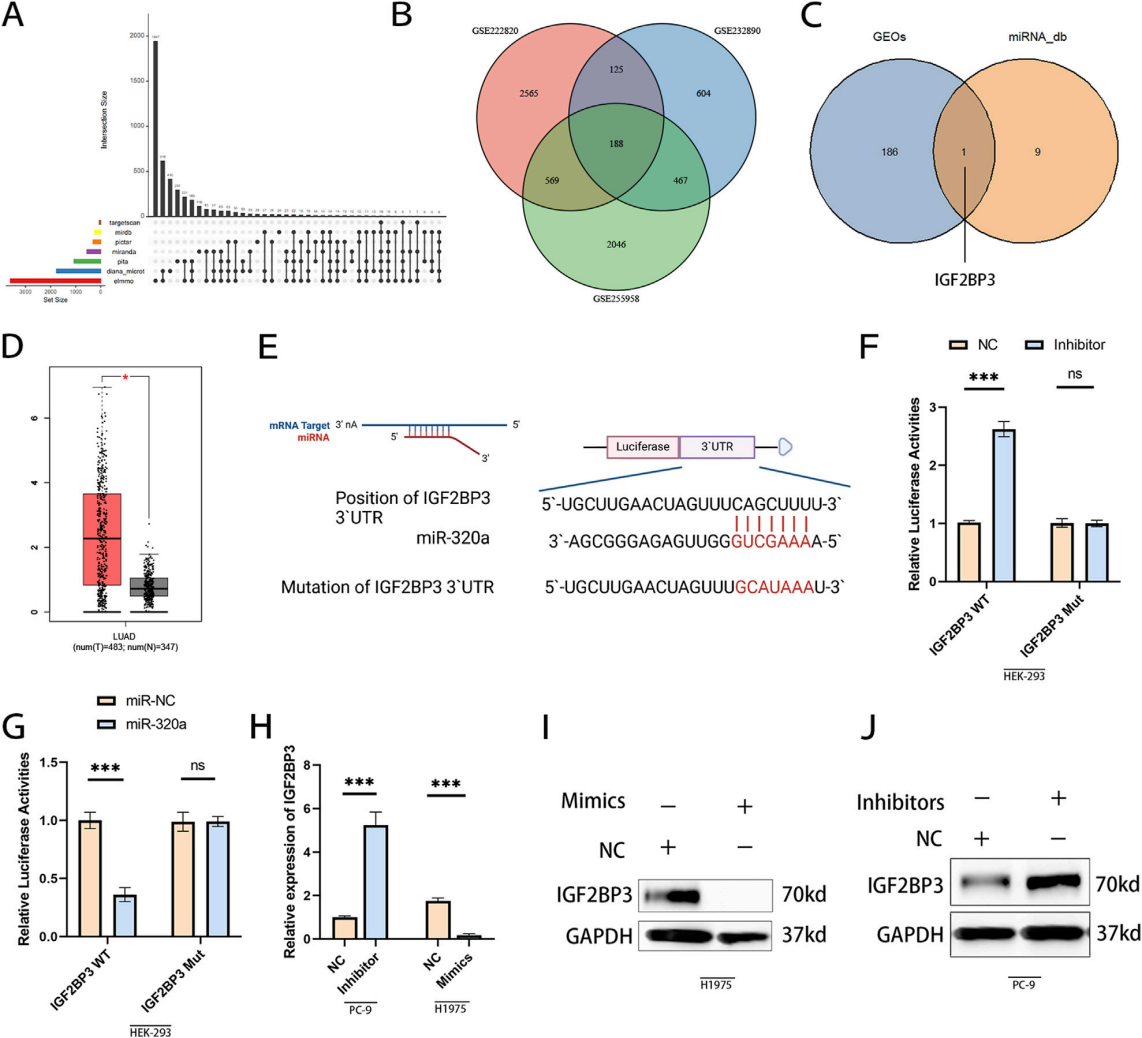
miR-320a directly targets IGF2BP3 to regulate osimertinib resistance. **(A)** Intersection analysis of predicted miR-320a targets across seven miRNA databases using UpSetR (v1.4.0). **(B, C)** Integration of database predictions with differentially expressed mRNAs from three osimertinib-resistant NSCLC cohorts (GSE222820, GSE232890, GSE255958), identifying IGF2BP3 as the top candidate. **(D)** TCGA-LUAD analysis confirming elevated IGF2BP3 expression in tumors compared to adjacent normal tissues. **(E)** Schematic of the conserved miR-320a binding site in the IGF2BP3 3′UTR. **(F)** Transfection of wild-type (WT) IGF2BP3 3′UTR with miR-320a inhibitor significantly increased luciferase activity, while mutation (Mut) of the binding site abolished this effect. **(G)** Transfection of WT IGF2BP3 3′UTR with miR-320a mimics suppressed luciferase activity, with no effect observed in Mut constructs. **(H)** miR-320a mimics downregulated IGF2BP3 mRNA levels, while inhibitors upregulated its expression. **(I, J)** IGF2BP3 protein levels were reduced by miR-320a mimics and upregulated by miR-320a inhibitors. *p < 0.05, **p < 0.01, ***p < 0.001 (Student’s t-test).

Building on the evidence that miR-320a interacts with the AGO2-mediated silencing complex, we further investigated its downstream mRNA targets. To systematically identify key regulators of osimertinib resistance, we integrated predictions from seven miRNA databases using UpSetR (v1.4.0), a specialized R package for visualizing complex set intersections, which resolved limitations of traditional Venn diagrams in analyzing six or more datasets (Figure 4A). Subsequent cross-validation with differentially expressed mRNAs from three independent osimertinib-resistant NSCLC cohorts (GSE222820, GSE232890, GSE255958) identified IGF2BP3 as the candidate (Figures 4B, C). TCGA-LUAD analysis confirmed elevated IGF2BP3 expression in tumors versus normal tissues (Figure 4D), consistent with its oncogenic role.

To further verified miR-320a directly targets IGF2BP3 via 3′UTR Binding. A conserved miR-320a binding site was predicted in the IGF2BP3 3′UTR (Figure 4E). Dual-luciferase reporter assays validated this interaction: miR-320a mimics significantly suppressed luciferase activity of wild-type IGF2BP3 (WT), while mutation of the binding site (Mut) abolished this effect (Figure 4F). Conversely, miR-320a inhibition rescued WT activity (Figure 4G), confirming sequence-specific regulation. WB showed miR-320a mimics downregulated IGF2BP3 mRNA and protein levels, whereas inhibitors markedly increased its expression (Figures 4H–J). These results demonstrated miR-320a as a tumor suppressor that directly suppresses IGF2BP3.

### The LINC01559/miR-320a/IGF2BP3 axis promotes osimertinib resistance *in vitro* and *in vivo*

**FIGURE 5:**
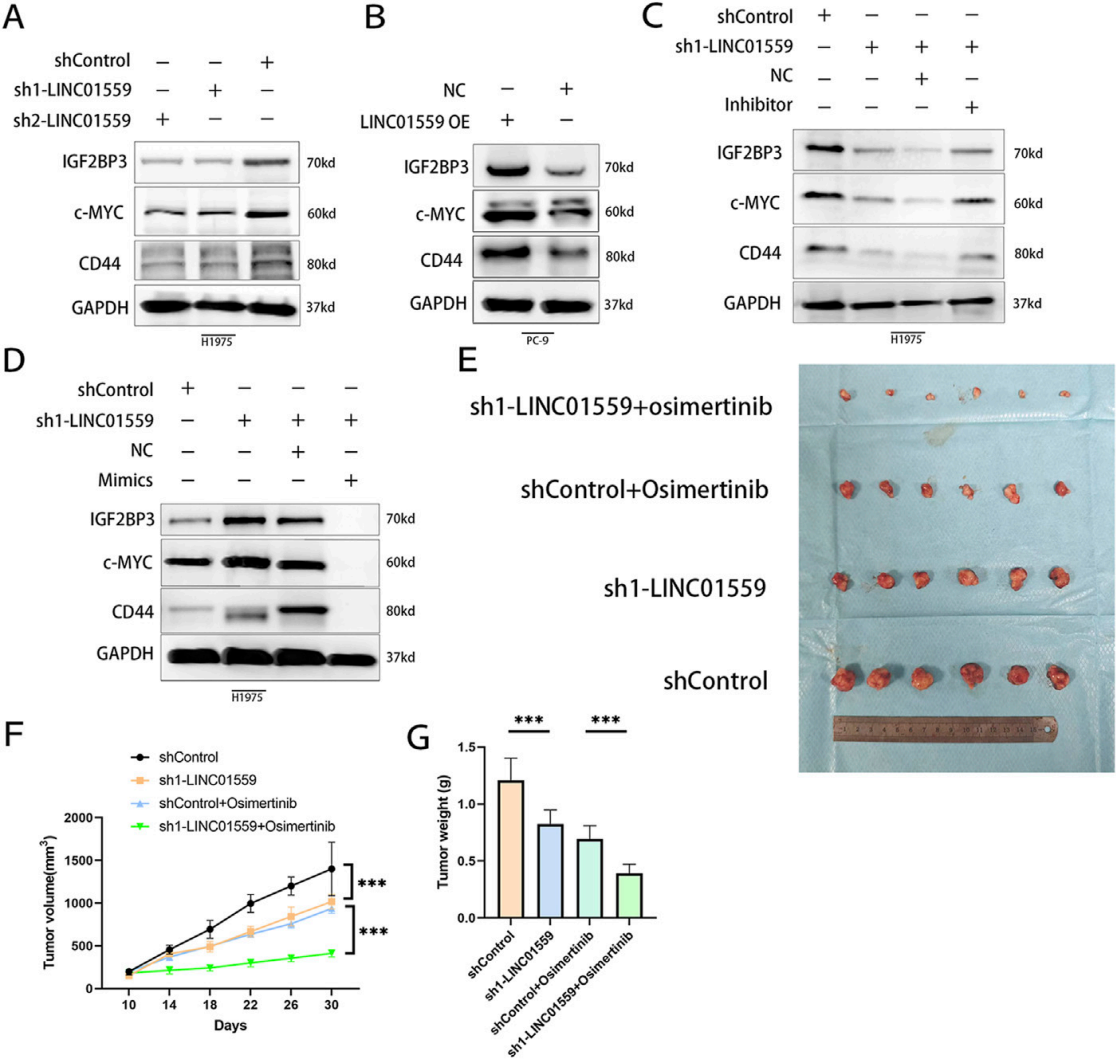
The LINC01559/miR-320a/IGF2BP3 axis drives osimertinib resistance *in vitro* and *in vivo*. **(A, B)** WB showing downregulation of IGF2BP3, c-MYC, and CD44 in LINC01559-KD cells and rescue by miR-320a inhibition. **(C, D)** LINC01559-OE upregulated these proteins, suppressed by miR-320a mimics. **(E–G)** Xenograft tumor growth curves and weights: LINC01559-KD combined with osimertinib synergistically suppressed tumor progression. Data are mean ± SD; *p < 0.05, **p < 0.01, ***p < 0.001.

To delineate the regulatory role of LINC01559 in the miR-320a/IGF2BP3 axis, we first examined the expression of IGF2BP3 and its downstream effectors, c-MYC and CD44, in LINC01559-modulated cells. Western blot analysis revealed that knockdown of LINC01559 significantly downregulated IGF2BP3, c-MYC, and CD44 protein levels in H1975 cells (Figure 5A), while overexpression markedly upregulated these oncoproteins in PC-9 cells (Figure 5B). Rescue experiments further validated the axis: inhibition of miR-320a partially restored IGF2BP3 and its downstream targets in LINC01559-knockdown cells (Figure 5C), whereas miR-320a mimics suppressed their expression in LINC01559-OE cells (Figure 5D).

To assess therapeutic relevance, we evaluated tumor growth in xenograft models. LINC01559 knockdown alone reduced tumor volume and weight compared to controls (Figures 5E–G). Strikingly, combining LINC01559 knockdown with osimertinib (sh1-LINC01559 + Osimertinib) synergistically suppressed tumor progression, with tumors exhibiting smaller volumes (Figure 5F) and reduced weights (Figure 5G).

**FIGURE 6:**
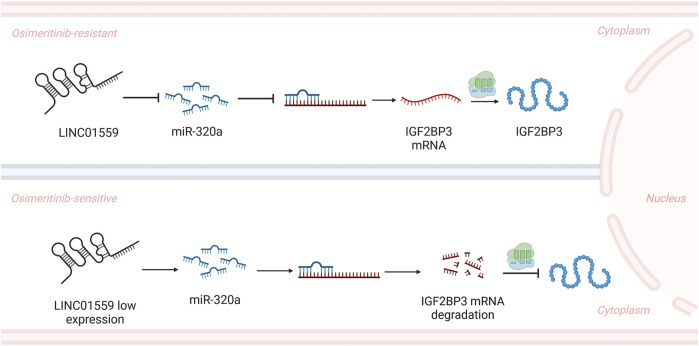
Schematic diagram of the entire study.

These findings collectively establish the LINC01559/miR-320a/IGF2BP3 axis as a critical driver of tumor growth and osimertinib resistance, highlighting its potential as a therapeutic target in NSCLC (Figure 6).

## Discussion

Our study characterizes LINC01559 as a clinically relevant lncRNA driving osimertinib resistance in lung adenocarcinoma. Analysis of multiple independent cohorts (GSE222820, GSE232890, GSE255958) and TCGA-LUAD data revealed consistent upregulation of LINC01559 in resistant tumors, with elevated expression correlating significantly with poor patient survival. Functionally, LINC01559 depletion sensitized resistant cells to osimertinib, suppressed colony formation, and attenuated migratory/invasive capacities, whereas its overexpression exacerbated therapeutic resistance. These results position LINC01559 as a central regulator of both aggressive phenotypes and drug adaptation, distinguishing it from other resistance-associated lncRNAs through its unique cytoplasmic localization and miRNA-dependent mechanism (Pal et al., 2022).

The cytoplasmic localization and miRNA-sponging activity of LINC01559 distinguish it from canonical lncRNAs implicated in tyrosine kinase inhibitor (TKI) resistance. For instance, nuclear lncRNAs such as MALAT1 predominantly regulate drug resistance through chromatin remodeling or ferroptosis inhibition (Huang et al., 2019; Shi et al., 2025), while HOTAIR recruits histone methyltransferases to silence tumor suppressor genes (Tsai et al., 2010). In contrast, LINC01559 operates primarily in the cytoplasm, functioning as a competitive endogenous RNA (ceRNA) to sequester miR-320a, thereby derepressing its target IGF2BP3. This spatial distinction underscores a novel regulatory layer in EGFR-TKI resistance, as cytoplasmic lncRNAs are less commonly linked to TKI adaptation compared to nuclear counterparts.

The LINC01559/miR-320a axis further expands the understanding of non-coding RNA crosstalk in TKI resistance by revealing a miRNA-dependent feedforward loop. Although previous studies have identified lncRNAs such as lncARSR and lncRNA-00742, among others, as miRNA sponge lncRNAs (Liao et al., 2020), LINC01559 uniquely couples miR-320a suppression with IGF2BP3 stabilization—a mechanism not previously reported in osimertinib resistance. IGF2BP3, an RNA-binding protein with oncogenic roles, is directly regulated by miR-320a, creating a synergistic axis that amplifies pro-survival signaling. Moreover, the LINC01559/miR-320a interaction highlights the functional versatility of miR-320 family members. For example, miR-320c has been shown to promote oxaliplatin responsiveness by targeting Chk1 (Lim et al., 2020), whereas miR-320a in this context drives drug resistance via IGF2BP3. This divergence underscores the specific roles of miRNA families in lncRNA-mediated pathways. By elucidating this axis, our study bridges a critical gap in understanding how cytoplasmic lncRNAs coordinate post-transcriptional regulation to sustain TKI resistance, offering a paradigm shift from nuclear-centric lncRNA mechanisms to cytoplasmic ceRNA networks.

IGF2BP3, a conserved RNA-binding protein with oncogenic properties, serves as a central mediator of LINC01559-driven osimertinib resistance. Analysis of TCGA-LUAD data revealed that elevated IGF2BP3 expression correlates with advanced tumor stages and poor patient survival, consistent with its established role in stabilizing pro-survival transcripts such as c-MYC and CD44 (Palanichamy et al., 2016; Liu et al., 2017). In our study, rescue experiments demonstrated that IGF2BP3 restoration reversed the tumor-suppressive effects of LINC01559 knockdown, confirming its functional indispensability in this axis. Mechanistically, LINC01559-mediated sequestration of miR-320a relieves post-transcriptional repression of IGF2BP3, allowing its accumulation in resistant cells.

IGF2BP3 stabilization fosters drug tolerance through regulation of mRNA stabilization. As an m6A reader, IGF2BP3 binds and stabilizes transcripts encoding oncogenic regulators, thereby amplifying survival signaling pathways that counteract osimertinib-induced apoptosis (Lu et al., 2024). This aligns with our observation that IGF2BP3 knockdown downregulated c-MYC and CD44, both critical for maintaining stemness and chemoresistance (Liu Y. et al., 2021). A recent study also demonstrate IGF2BP3 could binds internal mRNA m7G modifications, promoting transcript degradation via EXOSC2/exosome complex (Liu et al., 2024). Notably, IGF2BP3’s role in RNA stability extends beyond m6A modification. For instance, in endometrial carcinoma, IGF2BP3 interacts with lncRNA LINC00958 to stabilize E2F3 mRNA, driving tumor progression (Wang et al., 2022). Similarly, our study reveals that LINC01559-dependent IGF2BP3 stabilization creates a feedforward loop, where IGF2BP3 further enhances the stability of its own transcripts or other resistance-associated mRNAs. Therapeutic targeting of this axis is supported by preclinical evidence: combined LINC01559 silencing and osimertinib treatment cooperatively suppressed tumor growth in xenografts, suggesting that disrupting IGF2BP3’s RNA-stabilizing activity could re-sensitize resistant tumors.

The strong correlation between elevated LINC01559 expression and poor survival in LUAD patients positions it as a potential prognostic biomarker for osimertinib response prediction. This consistent with emerging efforts to integrate lncRNAs into precision oncology frameworks, such as MALAT1’s utility in stratifying NSCLC patients for adjuvant chemotherapy (Hou et al., 2023). Our TCGA analysis further supports LINC01559’s clinical relevance, as its overexpression coincides with advanced tumor stages and therapeutic relapse.

Preclinically, the synergistic efficacy of LINC01559 knockdown and osimertinib in xenograft models highlights the therapeutic promise of RNA-targeted strategies. Unlike conventional approaches focusing solely on EGFR inhibition, dual targeting of LINC01559 and osimertinib achieved marked tumor regression, suggesting that disrupting this axis could prevent adaptive resistance. However, clinical translation faces challenges, including optimizing lncRNA-specific delivery systems and minimizing off-target effects (Zhao et al., 2024). Innovations like lipid nanoparticle-encapsulated siRNA such as Patisiran or CRISPR-based lncRNA editing could overcome these hurdles (Adams et al., 2018; Davodabadi et al., 2024). Our findings advocate for further validation in patient-derived models to refine LINC01559’s biomarker utility and evaluate combinatorial regimens in osimertinib-resistant LUAD.

While our study establishes the LINC01559/miR-320a/IGF2BP3 axis as a driver of osimertinib resistance, we still have some limitations. First, the reliance on cell line-derived xenografts and TCGA data necessitates validation in patient-derived organoids or longitudinal cohorts to confirm clinical translatability. For instance, recent studies in NSCLC have employed organoid models to recapitulate TKI resistance dynamics (Lee et al., 2018), revealing microenvironment-dependent lncRNA functions that cell lines may not fully capture. Second, while we focused on miR-320a/IGF2BP3 as the primary axis, LINC01559 likely engages additional miRNAs or RNA-binding proteins to orchestrate resistance. Systematic approaches, such as crosslinking RNA immunoprecipitation (CLIP-seq) or lncRNA interactome profiling, could unmask these partners, as demonstrated in studies dissecting LINC00460s multi-target network in pancreatic cancer (Hou et al., 2021).

Future work should also explore the axis’s role in immune evasion, given IGF2BP3’s reported involvement in PD-L1 mRNA stabilization (Wan et al., 2022; Liu Z. et al., 2021). Additionally, spatial transcriptomics could resolve whether LINC01559 expression is enriched in tumor niches prone to osimertinib resistance. Finally, developing LINC01559-targeted therapies—such as antisense oligonucleotides or CRISPR interference—in combination with osimertinib warrants testing in immunocompetent models to evaluate both efficacy and immune modulation.

## Conclusion

This study delineates a novel LINC01559/miR-320a/IGF2BP3 axis driving osimertinib resistance in EGFR-mutant NSCLC. By integrating multi-omics data with functional validation, we highlight the therapeutic potential of disrupting this axis to overcome resistance.

## Data Availability

The datasets presented in this study can be found in online repositories. The names of the repository/repositories and accession number(s) can be found below: https://www.ncbi.nlm.nih.gov/geo/, GSE222820 https://www.ncbi.nlm.nih.gov/geo/, GSE232890 https://www.ncbi.nlm.nih.gov/geo/, GSE255958.
